# Speech, movement, and gaze behaviours during dyadic conversation in noise

**DOI:** 10.1038/s41598-019-46416-0

**Published:** 2019-07-18

**Authors:** Lauren V. Hadley, W. Owen Brimijoin, William M. Whitmer

**Affiliations:** Hearing Sciences – Scottish Section, Division of Clinical Neuroscience, University of Nottingham, Glasgow, UK

**Keywords:** Behavioural ecology, Human behaviour

## Abstract

How do people have conversations in noise and make themselves understood? While many previous studies have investigated speaking and listening in isolation, this study focuses on the behaviour of pairs of individuals in an ecologically valid context. Specifically, we report the fine-grained dynamics of natural conversation between interlocutors of varying hearing ability (n = 30), addressing how different levels of background noise affect speech, movement, and gaze behaviours. We found that as noise increased, people spoke louder and moved closer together, although these behaviours provided relatively small acoustic benefit (0.32 dB speech level increase per 1 dB noise increase). We also found that increased noise led to shorter utterances and increased gaze to the speaker’s mouth. Surprisingly, interlocutors did not make use of potentially beneficial head orientations. While participants were able to sustain conversation in noise of up to 72 dB, changes in conversation structure suggested increased difficulty at 78 dB, with a significant decrease in turn-taking success. Understanding these natural conversation behaviours could inform broader models of interpersonal communication, and be applied to the development of new communication technologies. Furthermore, comparing these findings with those from isolation paradigms demonstrates the importance of investigating social processes in ecologically valid multi-person situations.

## Introduction

Taking part in a conversation is a complex task that requires individuals to both comprehend the speech of a partner, and produce their own comprehensible speech. Conversing effectively requires quick alternation between these processes, with the intervals between turns (i.e. between playing a listening and a speaking role) often being under 250 ms^1^. The challenge is increased in noisy environments such as cafés or restaurants, which tax interdependent sensory and cognitive skills^2^. These situations are particularly demanding for people with hearing impairment (HI), and they often shun environments in which they may fail to keep up^3^. However, speakers and listeners can draw on a variety of behavioural strategies to aid communication in such environments. In this paper we investigate the strategies individuals use spontaneously when conversing with a partner in noise, in order to identify the strategies that are spontaneously employed to facilitate communication in such contexts.

In a conversation, the speaker’s aim is to convey information in an intelligible manner. Previous studies of talking in noise have shown that speakers do this by modifying the acoustic parameters of their speech, and their speech patterns, both when producing speech in isolation and when speaking in conversation. In terms of acoustic parameters, speakers in noisy environments increase their vocal intensity and adjust the spectrum of their speech^4,5^, which improve intelligibility for listeners^5,6^. In terms of speech patterns, speakers in noisy environments produce longer utterances^7^ and speak the utterances that they do produce more slowly (i.e., producing fewer syllables per second), thereby giving listeners more time to process spoken information^8,9^. Finally, it has been shown that in noise, speakers include more (and potentially longer) pauses^8^. While this could reflect a strategic adjustment to aid listener processing (and has been interpreted as such), it could alternatively be the result of missed turn-switches.

The listener’s aim in a conversation, on the other hand, is to comprehend the speaker’s message. This is facilitated by being able to hear the speaker better, or by receiving additional, non-auditory, cues conveying message content. To hear the speaker better, listeners can orient their ear to increase signal strength^10,11^, with best results when they turn 30 degrees away from the sound source^12^. Indeed listeners with unilateral hearing impairment have been found to adjust their head to increase the speech signal in adverse listening conditions, though they may be particularly aware of the impact of orientation on their hearing^13^. While highly variable, even normal hearing listeners have been shown to adjust their head movements when speech becomes difficult to hear^12^, though this may be reduced by competing visual information^11^, and few listeners reach an optimal orientation^12–14^. Decreasing the distance between interlocutors would also allow a listener to better hear a speaker by increasing the signal-to-noise ratio. In terms of non-auditory cues, listeners show remarkable consistency in directing their gaze toward an active speaker^15,16^, and experience benefit from seeing them while they talk. For example, seeing a speaker’s head movement improves speech intelligibility^17^, while seeing their lip movements improves both speech intelligibility^18,19^ and speech detection^20^. Hence visual cues provide valuable additional information for processing speech successfully.

It is clear that a variety of strategies are available to the speaker and listener experiencing difficulty in noisy environments. But strikingly, the majority of studies investigating speaking and listening strategies have removed the social context in which these behaviours most often occur. Studies taking this isolationist approach involve speakers producing scripted utterances for a microphone in an otherwise empty room^4,6,9^, and listeners being presented with such pre-recorded speech in a similarly desolate context^6,12,13,17,18,20,21^. It is notable that the behaviours that are used to facilitate interpersonal understanding are investigated in isolated, offline, paradigms. While such work provides insight into the strategies that people can use to facilitate speaking and listening in noisy environments, it is yet to be determined whether such strategies are used spontaneously in an interactive context. Several studies attempting to address this question have used multi-person paradigms with highly constrained information-sharing^5,7,8,19^, and have often focused on only one modality of behaviour, removing the possibility of investigating how strategies occur in combination.

**Figure 1:**
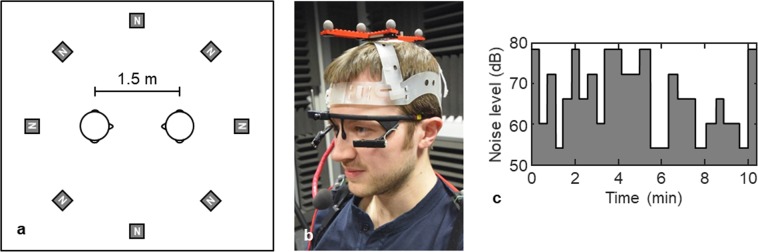
Experimental set-up (example of a non-participating individual). Panel a shows the participant setup within the sound attenuated room, showing the loudspeakers (N) presenting noise throughout each trial. Panel b shows the equipment setup including motion tracking crown, eye-tracking glasses and microphone. Panel c shows an example of the noise levels (54–78 dB in 6 dB increments) as a function of time during an example conversation trial.

In line with the broader shift toward addressing interaction using ecologically valid contexts that involve mutual adaptation^22,23^, we investigate dyadic conversation behaviour in dyads approximately matched in age and hearing loss, in noisy environments. Specifically, we focus on the speech, head movement, and gaze behaviours of people with varying hearing ability conversing without hearing aids in speech-shaped background noise fluctuating between 54 dB and 78 dB (see Fig. 1). We hypothesise that in higher levels of noise, speakers will increase their speech level and utterance duration, as well as increasing the duration of pauses between turns. We also hypothesise that listeners will orient their head to improve reception of the auditory signal (even if they do not reach the optimal 30 degree orientation), and that they will increase their gaze toward the talker - specifically the talker’s mouth. Finally, we anticipate that individuals will move toward each other to optimise exchange of information. By investigating the broad array of behaviours that HI individuals use while holding real conversations in different levels of noise, we extend prior work on individual speaking and listening to an interactive setting, and address how multiple strategies are used concurrently.

## Results

### Speech

**Figure 2:**
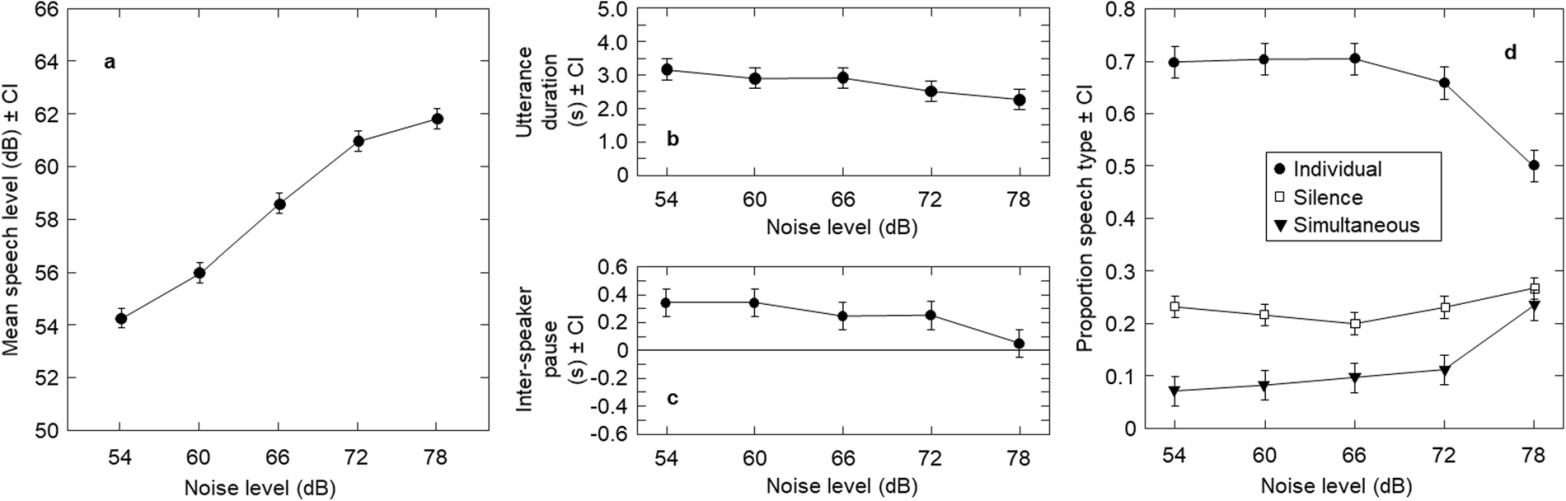
Speech adjustments by noise level. Panel a shows mean speech level by noise level. Panel b shows utterance duration by noise level, and panel c shows inter-speaker pause duration by noise level. Panel d shows proportion of time for individual speech, overlapping speech, and silence, by noise level. All error bars show 95% within-subject confidence intervals.

Average speech level significantly increased as noise level increased (F(2.075,60.182) = 271.72, *p* < 0.001, ηp2 = 0.90). Participants spoke on average 1.9 dB more loudly with each 6 dB increase in noise; i.e. they increased vocal level by 0.31 dB per 1 dB noise level increase (see Fig. 2a). Increasing noise level also led to significantly shorter utterances, (F(1.85,53.52) = 5.48, *p* = 0. 0.008, ηp2 = 0.16; see Fig. 2b), and significantly shorter median inter-speaker pauses (F(2.48,34.76) = 7.37, *p* = 0.001, ηp2 = 0.35, see Fig. 2c). The overall mean of these inter-speaker pauses was 247 ms, comparable to previous turn-taking results for English speakers (236 ms)^1^.

Noise level also affected conversational structure, showing an interaction with speech type – i.e., individual or overlapping (F(1.40,19.56) = 31.38, *p* < 0.001). There was a significant effect of noise level on both types of speech (individual: F(1.55,21.64) = 33.54, *p* < 0.001, ηp2 = 0.71; overlapping: F(1.36,19.03) = 22.10, *p* < 0.001, ηp2 = 0.61). Pairwise comparisons showed that that in comparison to all quieter levels, when background noise was at its loudest there was a lower proportion of individual speech (*p*s < 0.001), alongside a higher proportion of speech overlap (*p*s < 0.007). See Fig. 2d.

### Movement

**Figure 3:**
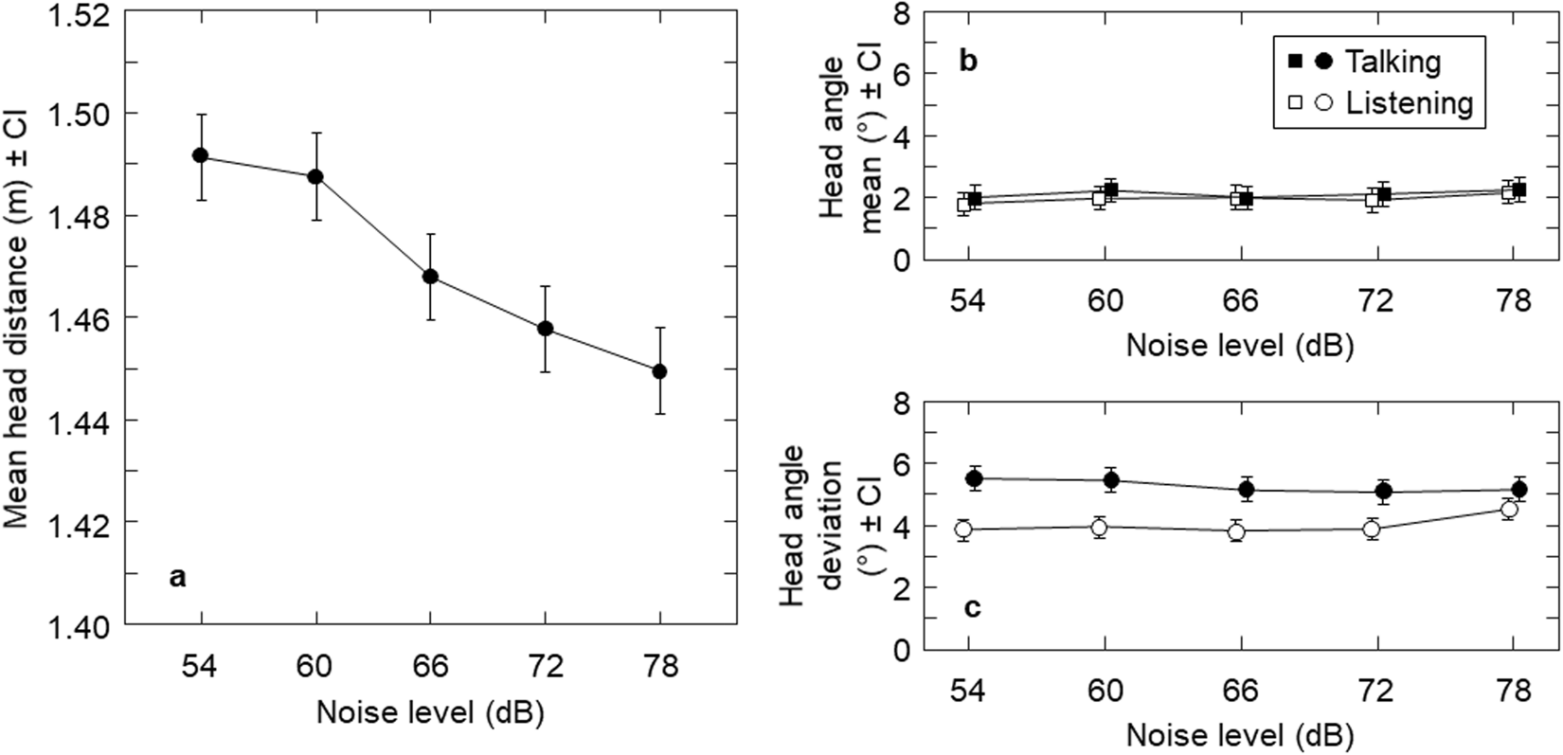
Movement adjustments by noise level. Panel a shows interpersonal distance by noise level. Panel b shows head (yaw) angle means, and panel c shows head (yaw) angle standard deviations, during periods of talking and listening by noise level. All error bars show 95% within-subject confidence intervals.

In terms of head position, interlocutors moved toward each other with increasing noise level (F(1.39,19.42) = 8.71, *p* = 0.004, ηp2 = 0.38; see Fig. 3a). On average, interlocutors decreased interpersonal distance by 10 mm for each 6 dB noise increase, equivalent to a 0.01 dB speech level increase per 1 dB noise level increase. Interlocutors showed a mean head angle of +2.1° from centre across conditions, indicating a slight turn of the left ear towards the partner, and listeners’ variability was affected by noise level (F(3.00,87.10) = 2.93, *p* = 0.04, ηp2 = 0.09; see Fig. 3c), with post-hoc tests showing a marginal increase between 54 dB and 78 dB (*p* = 0.07).

### Gaze

**Figure 4:**
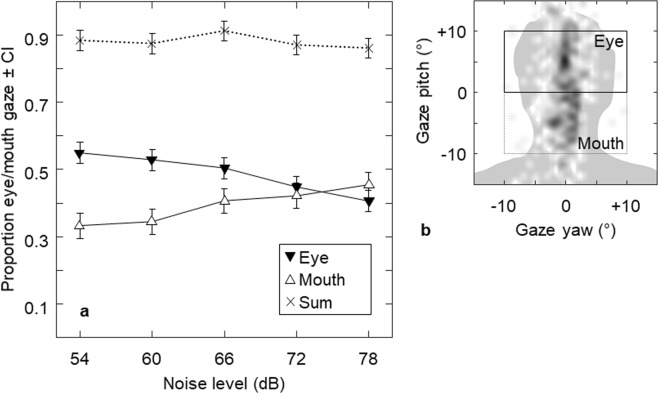
Gaze adjustments by noise level. Panel a shows proportion of listening time spent oriented toward the eye region, the mouth region, and the sum of the two, by noise level. Error bars show 95% within-subject confidence intervals. Panel b shows an example gaze pattern, with darker areas indicating more gaze time to illustrate how the gaze data was split into eye and mouth regions.

Listeners focused on their partner’s face (defined as 10° above to 10° below the height of the tragi, with a horizontal span of 20°) for an average of 88% of each trial (see Fig. 4). People spent a different proportion of time focused on the mouth (10° zone below the tragi) compared to the eyes (10° zone above the tragi) (F(1,116) = 8.38, *p* = 0.007, ηp2 = 0.22), and how much time they spent attending to the mouth vs eyes varied by noise level (F(4,116) = 11.92, *p* < 0.001, ηp2 = 0.29). As noise level increased, participants spent less time focused on their partner’s eyes (F(4,116) = 13.70, *p* < 0.001, ηp2 = 0.32) and more time focused on their partner’s mouth (F(4,116) = 7.38; *p* < 0.001, ηp2 = 0.20).

### Combination

If interlocutors start with an interpersonal distance of 1.5 m, and with each 6 dB noise increase they move 10 mm closer as well as speaking 1.9 dB louder. The combined acoustic benefit of these strategies amounts to 0.32 dB per 1 dB noise increase.

## Discussion

In this study, we measured speech parameters (such as speech level, turn duration, and inter-speaker pause), head movement, and gaze, to comprehensively investigate the strategies spontaneously used by individuals holding conversations in noisy environments. We have shown that while individuals employ potentially beneficial strategies during increased background noise (i.e. by increasing speech level and decreasing interpersonal distance), these adjustments only partially compensate for the increase in noise level. Indeed such behaviours amount to only 0.32 dB benefit per 1 dB noise increase. Other potentially beneficial strategies included using slightly shorter utterances, and increasing looks to the speaker’s mouth. While conversation structure remained constant until the noise level reached 72 dB, with minimal speech overlap and a high proportion of individual speech, a significant increase of overlapping speech at 78 dB suggests that at this level such strategies were not enough to avoid the turn-taking structure of conversation breaking down.

These findings demonstrate that the strategies people use during an interactive conversation are not the same as those used when speaking or listening in an empty laboratory, or even during an interactive task if it is highly constrained. For example, we did not find speakers to increase utterance duration with noise (which could indicate slower speech), as found in the interaction study of Beechey *et al*.^7^. Several possibilities could explain this difference. It is notable that the task given to our participants was relatively free, in comparison to a path-finding task, and so they may have chosen to change the content of their speech as opposed to slowing their production rate. Furthermore, Beechey *et al*. varied noise level with simulated environment, and these environments changed between, rather than within, trials. This design may have led participants to employ different strategies depending on the environment, rather than adjusting their use of strategies depending on noise level. The interesting prospect that strategy adjustment is based on noise level, while strategy selection is based on other parameters of the background noise, should be tested systematically in future.

Our data also showed inter-speaker pauses to shorten rather than lengthen. While shorter utterances may have simplified information processing for the listener, increasing pause duration would have provided further benefit. However, it is possible that prior findings of increased pausing in noise are a result of turn-switch difficulties, as opposed to being a strategy used to facilitate listener processing. Finally, we saw no use of head orientation to improve audibility, and report small changes in speech level and interpersonal distance. We suggest that this is because during an interactive conversation, interlocutors must deal with two conflicting goals: (1) facilitating communication, and (2) facilitating interpersonal connection. While strategies to achieve goal 1 have been addressed using isolation paradigms, goal 2 may mediate these strategies as well as eliciting other, purely social, behaviours. Hence interactive paradigms are essential to better understand natural conversation behaviours.

It is likely that while many behaviours reported in this study were used with the goal of improving communication, they may have been modified according to the social situation. For example, interlocutors did speak louder and move towards each other, but not enough to compensate for the background noise increase. Such apparent inefficiency could relate to the social inappropriateness of shouting to a conversation partner or invading another individual’s space. Head orientation strategies may have been avoided for similar reasons; since the optimal head orientation for audibility is 30°^12^, requiring listeners to turn their head somewhat away from their partner, it is possible that social constraints led individuals to avoid adjusting their head orientation. Alternatively, individuals may not have been aware of the SNR benefits of this strategy, and it is possible that with the noise surrounding the listeners any changes in speech-to-noise ratio elicited by re-orientation were not noticeable^24^. It should be noted, however, that listeners did increase their looks toward their partner’s mouth in higher background noise levels, potentially indicating prioritisation of the visual cues gained by looking directly to the mouth over the acoustic cues provided by turning the head.

While attempting to provide an ecologically valid conversation experience, the experimental situation may also have somewhat affected strategy use. The restriction that participants should not move the position of their chairs may have contributed to their minimal movement toward each other (although notably, chairs are often fixed in position). In addition, the use of speech-shaped noise may have masked the partner’s speech more strongly in the temporal domain than typical noises experienced in the background of everyday life (e.g., competing speech exhibiting envelope dips), reducing benefit from strategy use. Finally, the fact that conversing participants did not initially know each other may have impacted their behaviour; individuals may use different/better compensatory behaviours during conversations with familiar than unfamiliar partners. Yet while individuals may be comfortable to verbalise their difficulty when talking to familiar partners, it is perhaps most critical to understand what they do in situations when they are not; indeed daily life is full of conversations with unfamiliar interlocutors: from the postman to the barista. As it is clear that the behaviours that individuals spontaneously use while conversing in noise do not provide a high level of acoustic benefit, further work could investigate whether training could be implemented to allow individuals to take advantage of potentially useful strategies (such as learning to orient the head for maximal signal-to-noise benefit).

Future work could also begin addressing how conversation behaviours differ depending on the type of background noise, and how such behaviours are modified with increasing hearing impairment. In this study we used speech-shaped noise, and the constant masking may have made conversation particularly difficult. When listening against a background of other talkers, individuals may be able to ‘listen in the gaps’ to ameliorate difficulty, reducing reliance on facilitatory strategies. Furthermore, when participants do employ strategies, they may rely more strongly on those that increase signal-to-noise ratio to take advantage of dips in the masker (such as decreasing interpersonal distance or optimising head orientation). Investigating conversation behaviours in different sorts of background noise, such as babble, could be a valuable extension of this work. It is also important to note that this study was run with participants of varying hearing ability, centring around mild hearing loss. While this reflects typical hearing of individuals in the age range tested, a next step could be to investigate whether more severe hearing impairment leads to greater reliance on the strategies reported, or the uptake of new ones, as well as how strategy use is impacted by use of hearing aids.

This work highlights the importance of measuring social processes, and particularly listening behaviours, in multi-person contexts. By providing a comprehensive record of conversation behaviours across multiple modalities while engaged in challenging conversation situations, our findings could be used to hone models of interpersonal communication, for example addressing how visual and auditory cues are used simultaneously. These findings could also be exploited in new communication technologies to improve user benefit. For example, we show that gaze is well-focused on the partner, while head orientation is offset by several degrees. Such information indicates the potential value of taking gaze direction into account in hearing aid design^25^. The raw dataset is available as Supplementary Material for such purposes.

We have shown how people behave during real conversations in noise in stationary chairs, behaviour that differs notably from that occurring when speaking or listening in isolation. We report inefficient use of behaviours that have the potential to provide high levels of acoustic benefit (e.g., increasing speech level and decreasing interpersonal distance), as well as possible prioritisation of behaviours providing alternative benefits (e.g., shortening utterances and increasing gaze to toward a speaker’s mouth). We also show that individuals seemingly sustain conversation even in high levels of background noise (up to 72 dB), although an increase in overlapping speech indicates potential break-down of conversational turn-taking past this point. This work provides a first multimodal investigation of interactive conversation between individuals in noise, and is critically important for the field of communication technology. By understanding the strategies used by dyads conversing in challenging conditions, technological innovations can begin to include processing strategies that work with, rather than against, natural behaviours.

## Method

### Participants

Thirty unacquainted participants were divided into fifteen mixed-gender dyads (age_mean_ = 61 years, age_SD_ = 11 years; better-ear four-frequency pure-tone average (FFPTA across 0.5, 1, 2, and 4 kHz)_mean_ = 22 dB HL, FFPTA_SD_ = 12 dB HL). Within the available sample, participants were matched on age (difference_mean_ = 6 years, difference_SD_ = 5 years) and hearing asymmetry across ears (difference_mean_ = 3 dB HL, difference_SD_ = 2 dB HL). We also measured the difference in hearing loss between members of a pair (difference_mean_ = 7 dB HL, difference_SD_ = 6 dB HL). Each participant was paid £10 for taking part. This study was approved by the West of Scotland Research Ethics Committee (09/S0704/12). Methods were carried out in accordance with these ethical guidelines.

### Materials and task

Participants were seated in the centre of a ring of eight equidistantly spaced loudspeakers (Tannoy VX-6) in a sound attenuated room (4.3 × 4.7 × 2.6 m; see Fig. 1a). The loudspeakers each presented a different extract of steady-state noise with a spectrum equal to the long-term-average speech spectrum generated from data of Byrne and colleagues^26^, which includes recordings of male and female speakers across 12 languages. As noise levels in communal spaces are often over 70 dB^27,28^, we presented background noise continuously at 54, 60, 66, 72, or 78 dB, in 15–25 s segments with no gap between sequential levels. The complete counterbalancing of level ordering was determined using a paired de Bruijn sequence (individually sequenced for each trial^29^), and smoothing was applied for 10 ms between segments (see Fig. 1c). Each level was therefore presented five times, and hence each conversation lasted between 6 minutes 30 seconds and 10 minutes 50 seconds.

Vicon Tracker software was used to capture head-motion data, sampling at 100 Hz using a commercial infrared camera system (Vicon Bonita B-10 cameras fitted with wide angle lenses). Eight cameras were spaced around the room (one in each corner, plus one in the centre of each wall) to track 9-mm diameter reflective spheres that were arranged into uniquely identifiable ‘objects’ and attached to crowns on the head. Participant coordinates were measured in both Cartesian and polar space, calibrated to the centre of the floor. Temporal sampling rate was 100 Hz and spatial resolution was under 0.01°. Note that head position was recorded at the centre of the head (i.e. between the ears in line with the bridge of the nose) through reference to a pair of removable motion tracking goggles, as opposed to being recorded at the centre of the crown. Eye movement was recorded using 60 Hz Pupil Labs binocular eye trackers in 3D pupil detection mode and calibrated using a 5-point grid. The right eye was recorded in all participants except those that reported specific vision problems in that eye (two participants). Speech was recorded using a gooseneck microphone attached to the motion tracking crown approximately 6 cm from the participant’s mouth (see Fig. 1b).

The experiment was controlled using Matlab, which determined loudspeaker output, recorded motion capture data in Euler coordinates, and recorded eye angle data. Matlab was also used to trigger changes in the presentation level of the background noise by sending the requested level (dB SPL) in the form of an 8-bit integer. The Max/MSP visual programming language was used to receive and convert this trigger to dB, which controlled the playback of an 8-channel speech-shaped noise wav file. The first of these triggers also initiated the capture of speech signals from the microphones. All audio was run at 16 bits and 44.1 kHz sample rate, I/O was handled with a Ferrofish A-16 driven by an RME MadiFace XT on the host computer.

### Procedure

Participants were introduced and taken into a sound attenuated room and seated face-to-face at a distance of 1.5 m (they were asked not to alter chair position). The motion tracking crowns with lapel microphones attached via a gooseneck were then fitted. Participants then each put on a pair of eye-tracking glasses and were individually calibrated in front of a 92 × 74 cm monitor at a distance of 125 cm. Hearing aids were not worn during the experiment. In total, setup took approximately 40 minutes.

Each dyad held three conversations (i.e. three trials), each lasting approximately 9 minutes. The conversation topics focused on: films, close-call incidents, and the resolution of an ethical dilemma. In the film conversation^30^, participants were asked to discuss what they liked to watch. In the close-call conversation^31^, participants were asked to discuss a near miss: a time something bad almost happened but that worked out in the end. In the ethical dilemma conversation, participants were asked to come to a joint decision regarding the Balloon task^32^. In the Balloon task, participants must choose who to sacrifice from a descending hot air balloon between a scientist on the brink of curing cancer, a pregnant woman, and the pilot (her husband). Order of conversation topics was counterbalanced.

Participants were told that they should try to keep conversing the entire time that the experimenter was outside of the room, and that background noise would play from the surrounding speakers throughout. In between each conversation, the experimenter went into the room to confirm that participants were happy to continue, give them their next topic, and perform an eye tracker drift correction.

### Analysis

Data from one dyad was removed due to a motion tracking error (3 trials). Of the remaining 45 trials, a technical fault led to the loss of one audio recording, hence this trial was also removed. Analysis was conducted on the remaining 44 trials. Analyses were run using repeated-measures ANOVA on participant data averaged across all instances of each level across all conversation trials. Greenhouse-Geisser correction was used when assumptions of sphericity were violated (SPSS, v24). We also report partial eta squared values to indicate effect size (ηp2), for which a value of 0.01 is considered small and 0.26 is considered large^33^. Confidence intervals (95%) were calculated from the subject × condition interaction term^34^.

Periods of speech were detected from average root mean square (RMS) amplitude using an automated algorithm dependent on a manually-selected RMS threshold across a rolling window. A smoothing value of 0.1 Hz and a pause allowance of 1.25 s were used (due to few pauses being greater than 1.25 s in conversation)^35,36^. This allowed speech to be defined as periods during which an individual’s microphone recording was above threshold, and listening to be defined as periods during which the other individual’s microphone recordings were above threshold. Analyses of speech level were run only across the times that individuals were determined to be speaking, as opposed to over the entire recordings. Note that while microphones did pick up a small amount of the background noise, this amounted to a mean RMS level increase of only 0.39 dB for each 6 dB increase, and reported levels are corrected. Utterance duration was calculated across all speech segments with a duration of over 50 ms (to remove clicks). Any utterances that spanned a noise level transition were excluded from duration analyses.

Prior to analysis, eye tracking data was transformed to the Vicon axes (as opposed to the position to the eye camera) using the validation data from the start of the experiment. Drift was then corrected at the start of each trial through reference to the other participant’s head centre. Eye angle data was then added to head movement to generate gaze coordinates. Head angle and gaze angle were calculated in relation to the centre of the other participant’s head (i.e., oriented directly towards the other participant would be 0° pitch and yaw).

Anonymised data analysed during this study is included in Supplementary Information. Identifiable speech data is shared as binary data coding indicating when the recorded audio was over vs under threshold, i.e. when speech occurred.

### Informed consent

Informed consent was obtained from each participant prior to initiation of the study. Informed consent was also obtained for publication of images of non-participating individuals in an open-access journal.

## Supplementary information

All data

## Data Availability

Anonymised data analysed during this study is included in Supplementary Information. Identifiable speech data is shared as binary data coding when the recorded audio was over vs under threshold, i.e. when speech occurred.
